# Graphene Oxide increases mammalian spermatozoa fertilizing ability by extracting cholesterol from their membranes and promoting capacitation

**DOI:** 10.1038/s41598-019-44702-5

**Published:** 2019-05-31

**Authors:** Nicola Bernabò, Juliana Machado-Simoes, Luca Valbonetti, Marina Ramal-Sanchez, Giulia Capacchietti, Antonella Fontana, Romina Zappacosta, Paola Palestini, Laura Botto, Marco Marchisio, Paola Lanuti, Michele Ciulla, Antonio Di Stefano, Elena Fioroni, Michele Spina, Barbara Barboni

**Affiliations:** 1 Faculty of Bioscience and Technology for Food, Agriculture and Environment, University of Teramo, Via Renato Balzarini 1, 64100 Teramo, Italy; 2 Department of Pharmacy, University “G. d’Annunzio”, Via dei Vestini, 66100 Chieti, Italy; 3 School of Medicine and Surgery, University of Milano Bicocca, 20900 Monza, Italy; 4 Department of Medicine and Aging Sciences, University “G. d’Annunzio” Chieti-Pescara, 66100 Chieti, Italy; 5 Center on Aging Sciences and Translational Medicine (CeSI-MeT), University “G. d’Annunzio” Chieti-Pescara, 66100 Chieti, Italy; 6 Laboratorio Analisi Dr. Fioroni, Viale A. de Gasperi, 19, 63074 San Benedetto del Tronto, Italy

**Keywords:** Biomaterials - cells, Membrane biophysics, Biophysical chemistry, Biophysical chemistry

## Abstract

Graphene Oxide (GO) is a widely used biomaterial with an amazing variety of applications in biology and medicine. Recently, we reported the ability of GO to improve the *in vitro* fertilization (IVF) outcomes in swine, a validated animal model with a high predictive value for human fertility. For that reason, here we characterized the mechanisms involved in this positive interaction by adopting an experimental approach combining biological methods (confocal microscopy analysis on single cell, flow cytometry on cell populations and co-incubation with epithelial oviductal cells), physical-chemical techniques (Differential Scanning Calorimetry and Thermogravimetric Analysis), and chemical methods (mass spectrometry and lipid measurement). As a result, we propose a model in which GO is able to extract cholesterol from the spermatozoa membrane without causing any detrimental effect. In this way, the cholesterol extraction promotes a change in membrane chemical-physical properties that could positively affect male gamete function, modulating sperm signalling function and increasing in this way the fertilizing potential, without losing the ability to physiologically interact with the female environment. In conclusion, these data seem to suggest new intriguing possibilities in engineering sperm membrane for improving assisted reproduction technologies outcomes, even in human medicine.

## Introduction

The use of biomaterials has experienced an incredible dissemination for a myriad of applications, ranging from regenerative medicine to diagnostics. In this context, one of the most promising family of materials is graphene and graphene-related materials, whose use with biomedical purposes is continuously increasing. Graphene was first isolated in 2004 by Andre Geim and Konstantin Novoselov, awarded with the Nobel Prize in Physics in 2010 for its discovery. Graphene is a single layer of carbon packed in a hexagonal honeycomb lattice with carbon–carbon distances of 0.142 nm. Graphene possesses some interesting properties that it is interesting to note: it can be considered as an indefinitely large aromatic molecule, is the strongest material ever tested^1^, conducts very efficiently heat and electricity and is nearly transparent.

Immediately after its discovery, researchers started to develop a large family of graphene-related materials, such as few layer graphene sheets (FLGS), ultrathin graphite, graphene nanosheets, graphene oxide (GO), and reduced graphene oxide (rGO), with a wide range of applications in electronic, engineering, chemistry, and bio-medicine. In this last context, the hydrophilic forms of graphene have been used during the last years for several different applications including bio-imaging^2,3^, cancer theragnosis^4–6^, gene delivery^7^, tissue engineering^8,9^, biosensing^10^, DNA sequencing^11^ and drug delivery^12,13^.

Our attention was attracted by GO for two main reasons. First, it is a relatively hydrophilic molecule able to interact with biological structures in aqueous phase (as blood or other biological fluids). Moreover, the possibility that GO could exert a detrimental effect on reproductive function in some animal models had been suggested^14–16^, in particular when the spermatozoa are exposed to different GO forms^17–19^.

In our laboratory, for the first time, we assessed the effect of GO on mammalian spermatozoa in terms of fertilizing ability^20^. Sperm cells are unable to fertilize the female oocyte immediately after ejaculation and they only reach their fertilizing ability after residing for hours to days (depending on the species) within the female genital tract^21^. Here, and concretely in the oviduct isthmus, they complete their functional maturation (the so called capacitation) under the guidance of endocrine signals from the surrounding female environment^22^. Capacitation implies marked changes in sperm membranes, chemical composition and, as a consequence, on their physico-chemical properties. In particular, the presence of Ca^2+^ and bicarbonate activates a cAMP/PKA-dependent pathway that leads to the rupture of membrane asymmetry (inner and outer membrane leaflet are characterized by a different chemical composition) with the consequent exposure of cholesterol on the outer side of plasma membrane (PM)^23–33^. Once exposed, cholesterol is removed from the anterior area of sperm head by extracellular acceptors (albumin, globulins), thus promoting the increase in PM fluidity and the ability to fuse with the outer acrosome membrane (OAM) when the physiological stimulus, the oocyte zona pellucida (ZP), will be met^28,34^.

In our previous experiments, we found that boar spermatozoa co-incubation with GO under capacitating conditions was able to induce a dose-dependent effect. In particular, at relatively high concentrations (10 and 50 μg/ml), it was able to induce a toxic damage expressed as decreased viability and loss of acrosome integrity, while in a definite range of concentrations (0.5 to 1 µg/mL), surprisingly, GO seemed to promote the fertilizing ability in an *in vitro* fertilization (IVF) assay^20^. Since this unexpected effect could be interesting either for the understanding of the basics GO interaction with living systems as well as for the development of possible technological applications in assisted reproduction technologies (ARTs), here we carried out further experiments by combining biological, chemical and physical approaches, with the aim of exploring the molecular mechanisms of this interaction.

## Results

### GO affects sperm membrane chemical composition by reducing cholesterol concentration

To perform a complete molecular characterization of GO effect on membrane chemical composition, we checked the potential changes in a set of lipids known to be involved in biologically relevant processes, such as oxidative metabolism.

**Figure 1:**
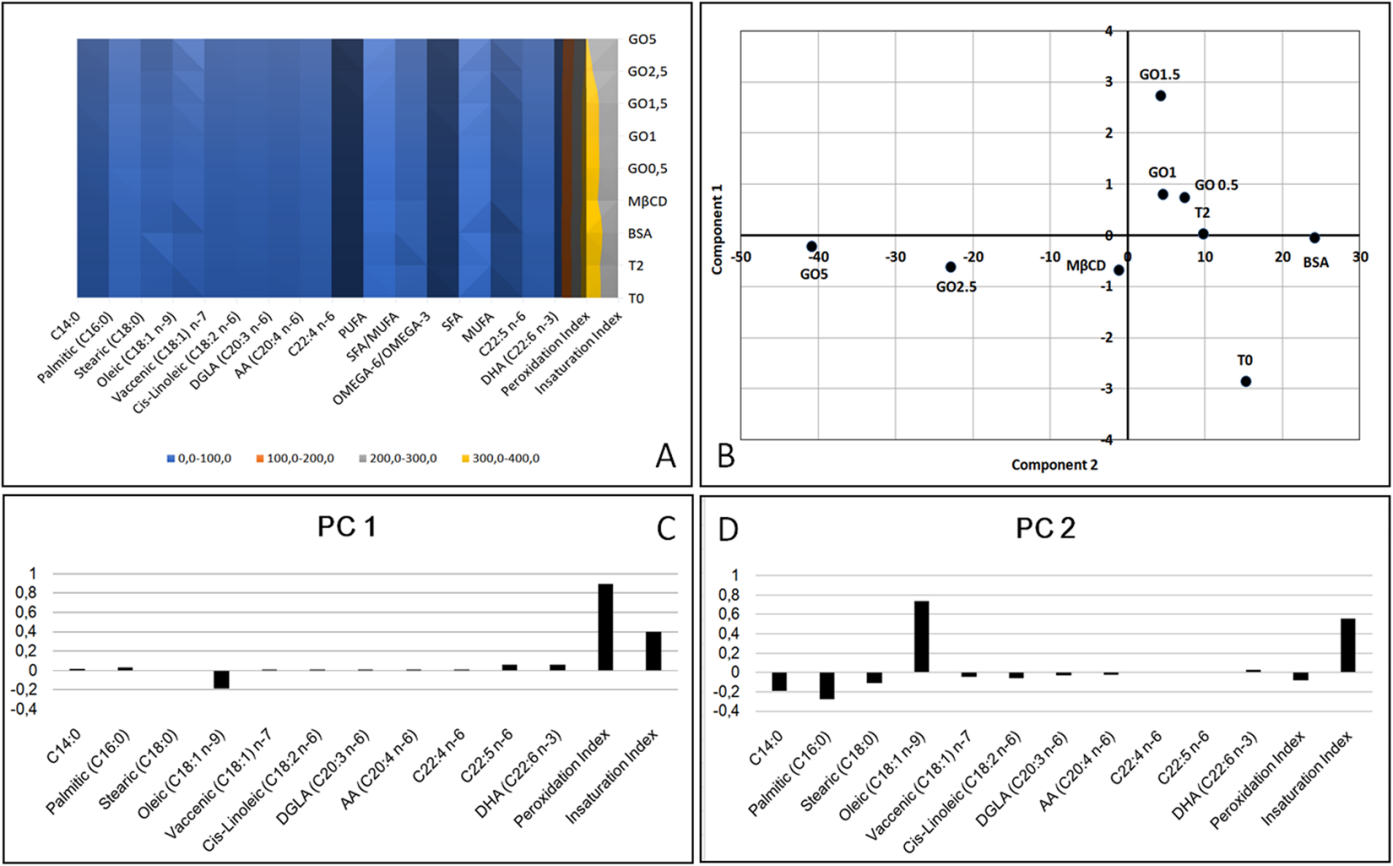
Effect of GO exposure on sperm membrane lipid composition. (Panel A) Effect of the exposure (in term of lipid composition) of boar spermatozoa to different GO concentrations, BSA and MβCD, compared to the membrane composition in physiological conditions before (T0) and after 2 h of incubation under capacitating conditions (T2). (Panel B) PCA analysis realized by assessing the different experimental treatments studied (different GO concentrations, BSA, MβCD). (Panel C) Histogram showing the values of PC1 with reference to all the lipids measured. (Panel D) Histogram showing the values of PC2 with reference to all the lipids measured.

As reported in Tables 1 and 2, it is evident that incubation of spermatozoa with GO under culture conditions causes some important modifications in the lipidomic pattern (see Supplementary Information 1 for further information). To infer such information, we adopted a multivariate approach based on PCA analysis, whose results are summarized in Fig. 1.

**Table 1:** Lipidomic analysis of control (T0 and T2) and treated (different GO concentrations, BSA, MβCD) samples. Values expressed as percentage (mean). (Panel B) Values expressed as percentage (standard deviation; SD).

Fatty Acids	T0	T2	H_2_O_2_	BSA	MBCD	GO 0.5	GO 1	GO 1.5	GO 2.5	GO 5
**(A)**										
C14:0	10,1	8,7	8,1	8,6	8,0	8,4	8,4	8,7	8,0	8,0
Palmitic **(**C16:0**)**	20,1	19,7	19,2	20,0	19,7	19,4	19,0	18,3	18,8	18,0
Stearic **(**C18:0**)**	10,0	9,7	10,0	10,1	10,6	9,8	10,2	9,5	10,2	10,2
Oleic **(**C18:1 n-9**)**	2,7	5,8	5,1	3,1	7,4	6,8	7,4	8,9	11,4	15,1
Vaccenic **(**C18:1**)** n-7	1,7	1,5	1,5	1,5	1,4	1,5	1,4	1,4	1,4	1,3
Cis-Linoleic **(**C18:2 n-6**)**	2,2	2,1	2,1	2,4	2,0	2,1	2,0	1,8	2,1	1,8
DGLA **(**C20:3 n-6**)**	1,5	1,5	1,4	1,4	1,4	1,4	1,3	1,3	1,4	1,2
AA **(**C20:4 n-6**)**	3,0	2,6	2,9	2,9	2,7	2,8	2,7	2,8	2,7	2,5
C22:4 n-6	1,1	1,1	1,1	1,2	1,2	1,1	1,1	1,1	1,0	1,0
C22:5 n-6	24,5	24,4	24,9	25,2	23,6	24,2	23,9	23,8	22,1	21,1
DHA **(**C22:6 n-3**)**	23,1	22,7	23,5	23,6	22,1	22,6	22,5	22,6	20,8	19,8
SFA	40,2	38,1	37,3	38,8	38,3	37,6	37,6	36,4	37,0	36,1
MUFA	4,4	7,4	6,7	4,6	8,8	8,3	8,8	10,3	12,9	16,4
PUFA	55,4	54,5	56,1	56,6	53,0	54,1	53,6	53,3	50,1	47,4
SFA/MUFA	9,2	5,2	5,6	8,5	4,4	4,6	4,3	3,6	2,9	2,2
OMEGA-6/OMEGA-3	1,4	1,4	1,4	1,4	1,4	1,4	1,4	1,4	1,4	1,4
Peroxidation Index	353,7	348,5	359,0	361,4	338,9	346,3	343,8	343,4	319,4	303,4
Insaturation index	235,5	235,0	241,1	240,6	230,1	234,4	233,2	234,2	221,5	214,5

**Table 2:** Lipidomic analysis of control (T0 and T2) and treated (different GO concentrations, BSA, MβCD) samples. Values expressed as standard deviation.

Fatty Acids	T0	T2	H_2_O_2_	BSA	MBCD	GO 0.5	GO 1	GO 1.5	GO 2.5	GO 5
C14:0	0.351	1.113	0.798	1.408	0.996	1.431	1.299	1.542	1.000	1.099
Palmitic (C16:0)	0.728	3.219	0.385	1.066	0.895	0.730	1.103	0.235	1.631	1.452
Stearic (C18:0)	0.979	2.760	1.228	1.430	0.650	0.992	0.837	0.593	0.863	0.876
Oleic (C18:1 n-9)	0.155	0.925	0.779	0.229	0.785	0.486	1.069	1.099	1.617	2.391
Vaccenic (C18:1) n-7	0.180	0.103	0.042	0.012	0.066	0.059	0.090	0.149	0.042	0.075
Cis-Linoleic (C18:2 n-6)	0.437	0.445	0.460	0.332	0.498	0.521	0.662	0.734	0.327	0.458
DGLA (C20:3 n-6)	0.289	0.297	0.131	0.102	0.153	0.169	0.229	0.263	0.173	0.184
AA (C20:4 n-6)	0.242	0.158	0.056	0.071	0.076	0.111	0.118	0.115	0.121	0.111
C22:4 n-6	0.085	0.321	0.111	0.176	0.304	0.112	0.085	0.101	0.036	0.032
C22:5 n-6	0.736	3.668	1.425	2.348	1.018	1.943	0.261	1.558	1.266	0.375
DHA (C22:6 n-3)	1.531	3.865	0.422	0.621	0.877	0.179	1.565	1.739	0.297	1.155
SFA	1.078	6.764	2.407	3.419	2.085	3.013	1.465	2.095	2.482	2.087
MUFA	0.100	0.984	0.760	0.217	0.813	0.512	1.056	1.094	1.595	2.349
PUFA	0.989	7.627	1.742	3.559	1.424	2.993	0.590	2.912	1.490	0.276
SFA/MUFA	0.452	0.465	0.921	0.602	0.652	0.489	0.664	0.314	0.570	0.487
OMEGA-6/OMEGA-3	0.155	0.121	0.116	0.089	0.141	0.114	0.152	0.153	0.105	0.139
Peroxidation Index	10.0	53.1	6.6	20.2	5.9	14.7	10.2	19.1	6.2	5.9
Insaturation index	6.0	33.3	5.4	13.4	4.8	10.0	6.0	11.2	5.2	2.7

In particular, it is evident that after 2 h of incubation the samples incubated under control conditions (T2) and those treated with the lower concentrations of GO (GO 1 and GO 0.5) are similar, while BSA and MβCD are characterized by more specific features. Interestingly, at higher GO concentrations, it is possible to hypothesize a dose-dependent effect in term of lipid modifications.

Looking at the PCA, it is feasible to identify the contribution of peroxidation index, unsaturation index and oleic acid as main contributors in variance, as well as the differences among the lipid components in different samples (see Supplementary Information 2). Oleic acid is particularly important in the spermatozoa as an energy provider for the acrosome reaction and it is associated to the induction of hypermotility^35^. Therefore, it is understandable to witness an increase in this fatty acid during capacitation. However, the relation between this and the concentration of GO is still unclear. The peroxidation index reports how susceptible the membrane is to peroxidation since only PUFA (i.e. poly-unsaturated fatty acid) can be peroxidated^36^. An increase in this index is a likely indicator that the membrane is not suffering from oxidative stress. Furthermore, contrary to what could be expected, higher levels of unsaturation have been related to better quality semen in humans and, in particular, it has been associated to higher levels of motility^37^.

In addition, since change in cholesterol and phospholipids ratio on sperm membrane is a key controller of sperm function, we assessed the effect of GO exposure on this parameter, in comparison with the effect produced by two other molecules known to influence this balance (i.e. BSA and MβCD).

**Figure 2:**
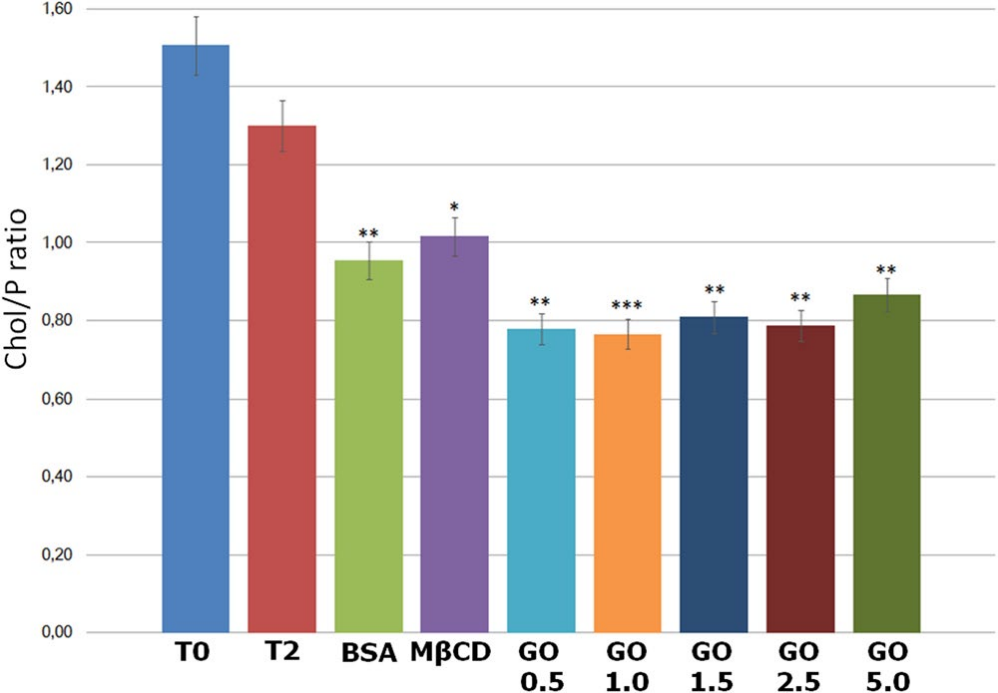
Cholesterol/Phospholipids ratio in sperm samples. Data are refered to the different experimental treatments. The asterisks denote statistically different group of data.

As expected, the incubation under capacitating conditions without cholesterol acceptors for 2 h did not produce any statistically relevant effect on cholesterol removal and consequently, the cholesterol/phospholipids ratio remains unaffected. On the contrary, the presence of acceptors (BSA and MβCD) in the capacitation buffer caused a significant reduction of this parameter. It is very interesting that GO exhibits a similar effect, promoting the extraction of cholesterol in a dose dependent way, reaching the maximum effect at 1 μg/mL (Fig. 2).

### GO exposure changes sperm membrane chemical-physical properties

Different parameters related with sperm function that are influenced by membrane composition were assessed, in particular the effect of GO on membrane fluidity (FRAP analysis and DSC), membrane potential and ions permeability (both by flow cytometry).

**Figure 3:**
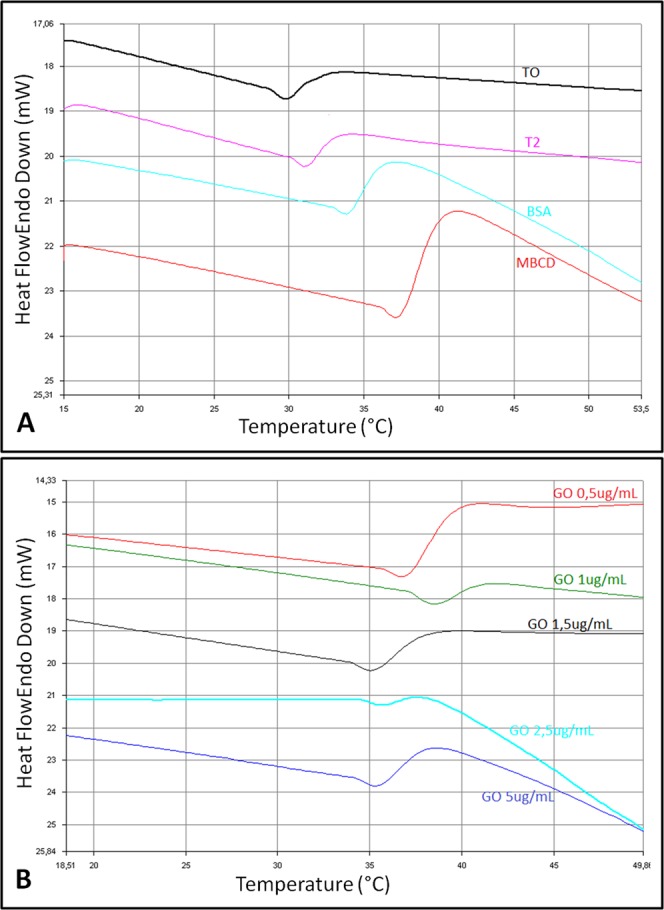
Differential Scanning Calorimetry (DSC) analysis. Different T_m_ corresponding to the sperm groups under study (treated with different GO concentrations, BSA or MβCD).

DSC analysis revealed substantial differences among the membrane thermograms. Figure 3 shows endotherms corresponding to thermotropic phase transitions recorded during heating from 15 to 90 °C. A narrowing of the transition peak was observed for the sample T2 (after incubation for 2 hours) when compared to T0 (before incubation). This effect is greatest when spermatozoa are incubated with MβCD and GO 1 µg/mL. The cholesterol ratio affects also the transition peak temperature (T_m_) of the samples, which increases as the concentration of cholesterol decreases.

In particular, an increased T_m_ was observed for samples co-incubated with the cholesterol acceptors BSA and MβCD, as well as in the presence of GO (Table 3). On increasing GO concentration, T_m_ shifted to lower temperatures in an approximately dose-dependent manner, indicating that the capacity to extract cholesterol is reduced when increasing the GO concentrations. The highest value of T_m_, higher than that recorded for BSA and MβCD enriched samples, was observed for the lowest concentrations of GO (0.5 and 1 µg/mL).

**Table 3:** Melting temperatures (T_m_) expressed in °C of the different samples are listed.

Mean	Onset (°C)	Peak (°C)	End (°C)	Peak width	ΔH (J/g)	Peak Height	Area (mJ)
T0	31,8	34,9	46,0	14,2	−11,8	−1,0	−46,1
T2	35,2	38,0	47,8	12,6	−26,3	−1,1	−56,5
BSA	34,3	37,9	53,2	18,9	−33,8	−1,5	−104,6
MβCD	34,5	37,6	50,3	15,8	−44,3	−1,5	−93,0
GO 0,5	36,1	38,9	44,2	8,1	−11,8	−1,2	−35,4
GO 1	35,1	37,4	43,5	8,4	−16,6	−0,8	−29,1
GO 1.2	36,0	38,4	48,1	12,1	−4,1	−0,6	−7,1
GO 2.5	35,7	38,2	41,9	6,1	−5,9	−0,7	−20,8
GO 5	35,5	38,7	45,8	10,2	−8,0	−1,0	−38,0
**St. Dev**.	**Onset** **(°C)**	**Peak** **(°C)**	**End** **(°C)**	**Peak** **width**	**ΔH (J/g)**	**Peak** **Height**	**Area** **(mJ)**
T0	1,6	1,3	5,5	6,9	4,1	0,5	10,0
T2	4,5	4,5	6,1	6,4	42,5	0,7	55,3
BSA	2,9	2,6	7,4	8,8	30,0	0,5	70,0
MβCD	2,5	2,5	8,5	9,4	57,7	0,6	73,7
GO 0,5	1,8	1,6	0,7	1,1	0,4	0,1	1,2
GO 1	2,9	3,0	6,2	6,5	30,1	0,6	42,4
GO 1.5	0,7	0,6	4,6	5,3	4,3	0,5	0,1
GO 2.5	0,2	0,7	3,7	4,0	7,5	0,6	23,1
GO 5	0,3	0,0	1,3	1,5	0,5	0,3	5,1

**Figure 4:**
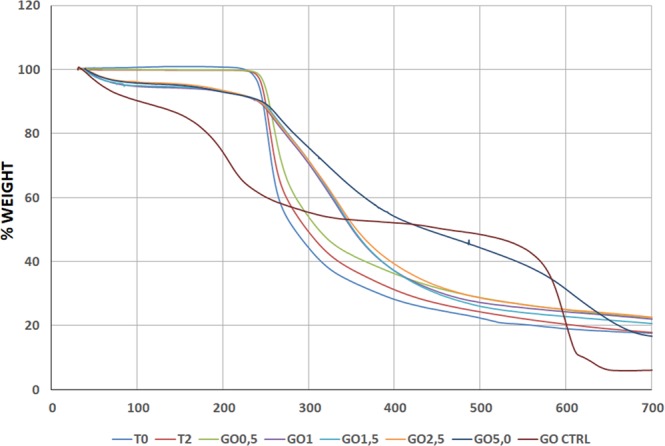
Thermogravimetric Analysis (TGA). Thermogram shows the differences between the sperm samples treated with the different experimental treatments (different GO concentrations, BSA or MβCD).

These results were confirmed by TGA analysis, that highlighted a behavior associated to the presence of GO in the sperm samples. In particular, the initial 4% weight loss at about 100–110 °C may be assigned to the release of CO_2_, CO and steam (water vapors). Since this weight loss is observed only in the GO enriched samples, except for 0.5 GO, it is evident that this loss is connected with the presence of GO. The following step (weight loss 4%) at 200 °C was assigned to the thermal elimination of labile oxygen-containing functional groups (i.e. hydroxyl, epoxy). A further gradual weight loss was observed for GO samples above 500 °C, corresponding to the elimination of more stable oxygen functionalities. In the temperature range from 350–500 °C there is a plateau in the GO samples curve, while the weight loss for pure spermatozoa is huge. At 350 °C it is possible to evidence a different behavior of weight loss for samples enriched at various GO concentrations. In particular, samples with a higher concentration of GO showed a reduced weight loss and an improvement in thermal stability, mainly attributable to the effect of physical barrier of GO nanosheets (Fig. 4).

**Figure 5:**
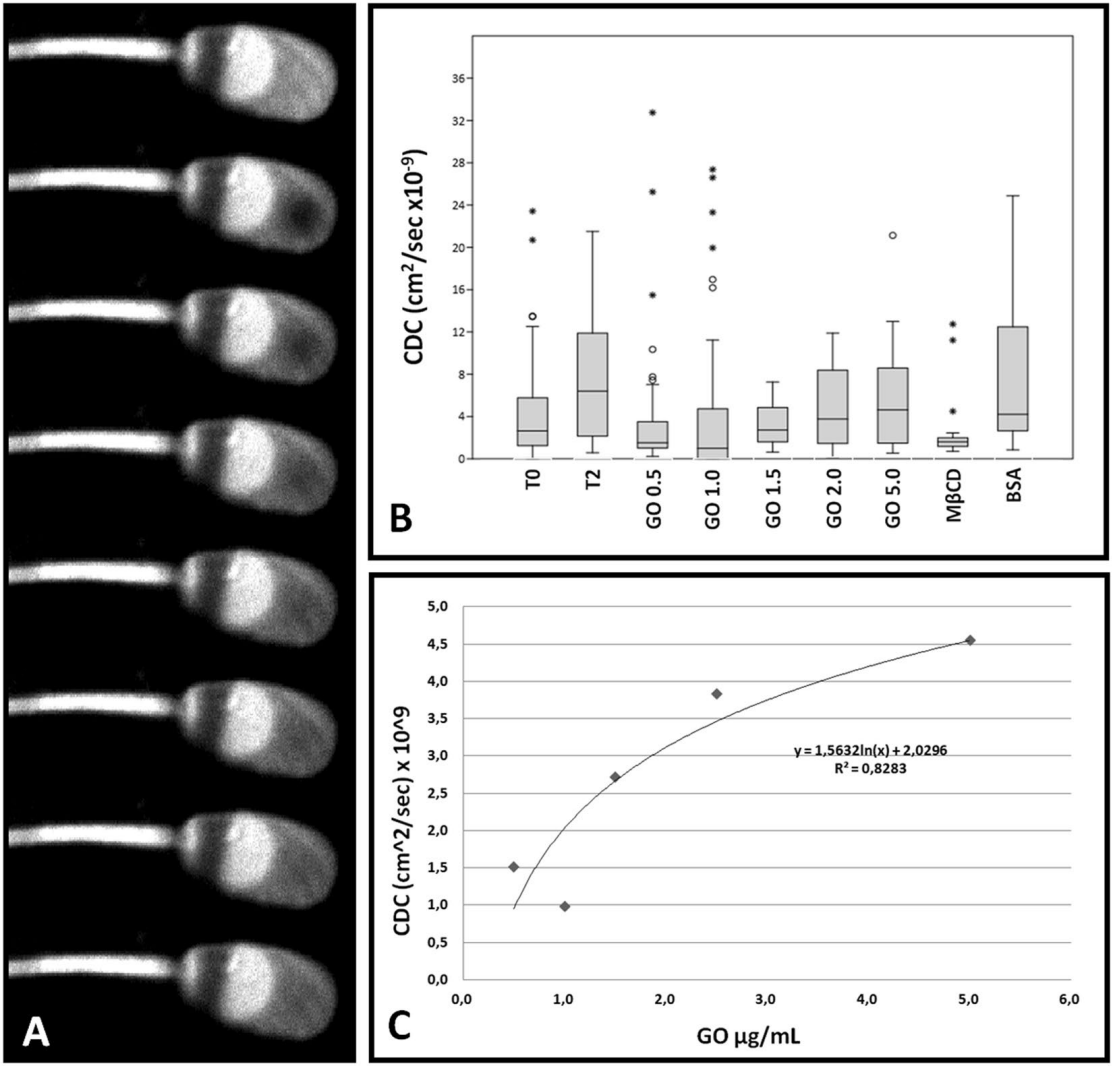
FRAP analysis of sperm cells. (Panel A) Exemplificative gallery showing the fluorescence recovery after photobleaching (FRAP) technique. (Panel B) Graph representing the values of Calculated Diffusion Coefficient (CDC), expressed as cm^2^/sec × 10^−9^, of sperm samples treated with the different experimental treatments (various GO concentrations, BSA or MβCD). The box plot represent the mean, 25°–75°, 5–90° percentile and out-layers. (Panel C) Dose-dependence linking GO concentration to Calculated Diffusion Coefficient (CDC).

The results of FRAP analysis on one hand confirmed our previous findings^20^ and on the other hand evidenced the dose-dependent effect of GO (R^2^ = 0.828) in the membrane fluidity increase (Fig. 5 and Supplementary Information 3).

### GO affects spermatozoa signal transduction

**Figure 6:**
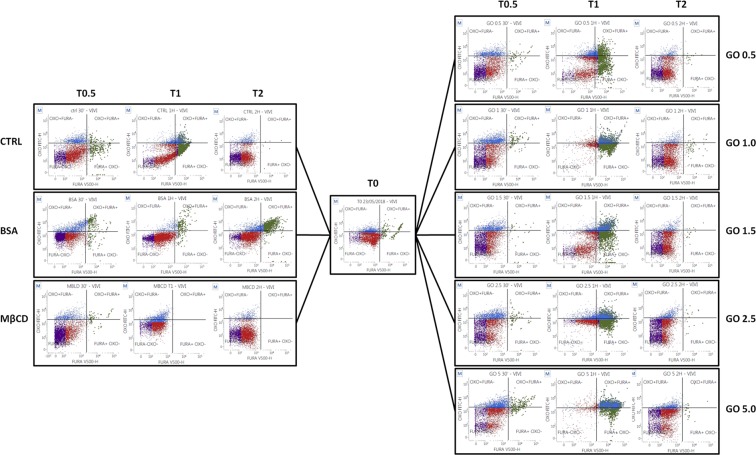
Flow cytometry results. (**A**) CTRL, BSA and MβCD samples at different times. Spermatozoa were stained with Fura 2-AM (x-axis) and Bis-oxonol (y-axis). (**B**) GO treated samples at different times. Spermatozoa were stained with Fura 2-AM (x-axis) and Bis-oxonol (y-axis).

Flow cytometry analysis of spermatozoa incubated with MβCD, BSA, or GO at different concentrations displayed no effect on membrane potential (Bis-Oxonol) when compared with control groups, while GO is able to influence the intracellular calcium concentration ([Ca^2+^]_i_) (Fig. 6A,B). Particularly, after 30 min and 1 hour of incubation an increase in the population marked positive for high [Ca^2+^]_i._ was noticed in every sample treated with GO, independently of the concentration. This fact suggests a possible interaction between GO and calcium channels by an unknown mechanism different to the change in the overall membrane potential, as it is evident due to the lack of signal of Bis-Oxonol.

### Effect of GO on sperm-SOEC interaction

**Figure 7:**
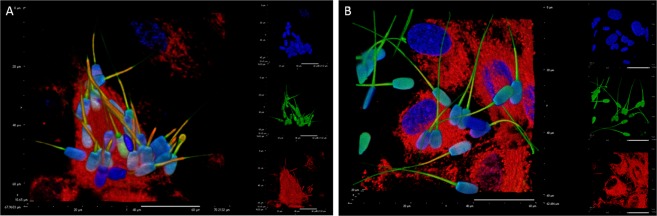
Confocal image showing the interaction of live spermatozoa with swine oviductal epithelial cells. Blue stain: Hoechst 33342 (nuclei); Red stain: DilC12 (membranes); Green stain CFDA (living spermatozoa).

We found no differences regarding the physical interaction of sperm cells with SOECs after pre-incubation with GO. As showed in Fig. 7, spermatozoa interact differently within the plate of cultured cells, evidencing a particular affinity to some cells compared to others. However this affinity remains unchanged in the presence of GO, indicating that preliminary incubation with GO does not interfere with the molecular mechanisms of subsequent sperm-oviduct interaction.

## Discussion

In a previous manuscript from our group it was demonstrated that GO, in a definite range of concentrations (0.5 to 1 µg/mL), is able to affect boar spermatozoa function leading to an increase in their fertilizing ability^20^. Since this finding was potentially applicable to ART, here we carried out a chemical and functional characterization of the GO interaction with swine sperm cells to explore the molecular mechanisms involved, with the aim to propose new strategies effective in control of sperm function in ART. As our previous data suggested a direct interaction between GO and sperm membrane, without entering into the cells, the approach was mainly focused in characterizing the effect of GO on sperm membranes. Importantly, sperm membranes are not only the interface with the external environment but also the key players in biochemical signalling involved in the acquisition of fertilizing ability^28,38^. Indeed, male gametes are unable to synthetize new molecules and their cytosol is virtually absent, so the main site of signal integration is the membrane, which is the key player of the capacitation process.

Mammalian spermatozoa, immediately after ejaculation, are unable to fertilize the female gamete, the oocyte, while they gain the full fertilizing ability only after they reside from hours to days (depending on the species) within the female genital tract. Here, it takes place a complex dialogue between male gametes and female organism (the capacitation) and spermatozoa became fertile. This process begins with the alteration of membrane physicochemical characteristics (the so-called “lipid remodelling”) (Brewis and Gadella, 2010; Gadella *et al*. 2008; Gadella and Luna, 2014; Bernabò *et al*. 2015). More in detail, the sperm plasma membrane (PM) and the outer acrosome membrane (OAM) markedly change their architecture and become able to fuse at the time of the acrosome reaction (AR), when the oocyte is reached. In lipid remodelling the cholesterol removal from PM has a pivotal role, indeed it is able to induce the increase in membrane fluidity, isotropy, and finally to allow the PM and OAM fusion (membrane fusogenicity) (Bruckbauer *et al*., 2010; Gadella *et al*. 2008; Kotwicka *et al*. 2011; Venditti and Bean, 2009).

The capacitation process is actively modulated by a complex functional dialogue with the oviductal environment, with the active participation of epithelial components that respond to the neuro-endocrine axis of female^39,40^.

Based on this biological background, we analysed the changes in the membrane chemical composition (lipidomic analysis, measurement of cholesterol/phospholipids ratio) to explore the molecular events related to the interactions of GO and sperm membrane. In particular, the physical-chemical properties (FRAP analysis, DSC analysis and TGA), the possible effect on signalling-related biochemical events (membrane potential, intracellular calcium concentration) and the interactions with swine oviductal epithelial cells (SOECs), were assessed. In all the experiments, the effects exerted by GO were compared to those exerted by serum album (bovine serum albumin, BSA), which is a physiological extracellular acceptor of cholesterol used to promote *in vitro* capacitation of swine spermatozoa, and methyl-β-cyclodextrin (MβCD), a stronger cholesterol extractor used for membrane engineering^41–43^.

First, we analysed the effect of GO during sperm incubation under capacitating conditions on sperm lipidomic pattern. In particular, as a functional parameter, we assessed the cholesterol/phospholipids ratio, since it is a key controller of the process that leads spermatozoa to the acquisition of their fertilizing ability. As it is well known^25,27,44^, the incubation of spermatozoa with bicarbonate and calcium is able to activate several cAMP/PKA-dependent pathways that lead to the activation of scramblase. That results in the translocation of cholesterol from PM of the acrosome area to the outer membrane leaflet and its consequent extraction by external acceptors. Since boar sperm membrane is characterized by a very high concentration of PUFA, the loss of cholesterol causes the increase in membrane isotropy and fluidity (collectively defined as “fusogenicity”), necessary to acquire the ability to undergo acrosome reaction when the ZP of the oocyte is met. Interestingly, in our experiment, GO was able to extract cholesterol with an efficiency similar to BSA and MβCD (p < 0.05 vs. CTRL, p > 0.05 among treatments), except in the case of 1 μg/ml, which showed a higher efficiency (p < 0.01 vs. CTRL). It is very suggestive that in our previous work this concentration of GO was able to promote the maximum effect in increasing the IVF efficiency, allowing us to hypothesize that cholesterol extraction mediated by GO might be one of the causes for the positive effect observed on sperm capacitation. This result confirms our previous hypothesis^20^ and is supported by recent *in silico* and *in vitro* studies^45,46^.

The effect of cholesterol extraction exerted by GO on sperm membrane physico-chemical behaviour was further characterized by physical (DSC) and biophysical (FRAP) approaches. DSC experiments gave us important information. The incubation under capacitating condition, as expected, promoted a detectable effect in terms of phase transition behaviour, when compared to T0 samples. The co-incubation with cholesterol acceptors (BSA and MβCD) was able to promote a very evident and different effect on T_m_ parameters, with the values shifted to higher temperatures in relationship to a reduction of the cholesterol concentration (see Table Tm and Fig. 3), in agreement with the tendency of cholesterol containing membrane to reduce the transition temperature^47^. This behaviour reflects the cholesterol-removal capacity of BSA, MβCD and GO at low concentrations. Indeed, treated spermatozoa with GO concentrations at 0.5 displayed a phase transition peak behaviour similar to that we found in MβCD-treated sperm cells, while at 1 µg/mL the peak is even higher, thus confirming the effective action of GO and strengthening the functional relevance of this treatment.

Higher concentrations of GO shift down the samples transition peak temperatures, indicating that the capacity of GO to extract cholesterol decrease in a dose-dependent manner (Table 3).

To better confirm this datum at the single cell level, we carried out a FRAP experiment that completed our previous analyses^20^. As it is evident (Fig. 5), the effect of GO in term of changes in membrane fluidity (expressed as Calculated Diffusion Coefficient, CDC) was different from that induced by BSA and MβCD and was in a dose-dependent manner (R^2^ = 0.828). These findings were finally confirmed by DSC experiments, which demonstrated the GO ability to modulate the membrane physical properties. This could likely be due to the membrane composition, since previous studies show that an increase in the membrane sterols content leads to a broadening of the transition peak^48^, evident in GO at 1 µg/mL (considering that this is the sample with the lowest cholesterol content). Furthermore, the decrease in the transition temperature is associated with an increase in mono-unsaturated fatty acids and specifically in oleic acid content, known to reduce the phase transition temperature and enlarge the peak^49^. Once again, our data are strengthening the dose-dependency and the specificity of GO interaction with sperm membranes.

From a functional point of view this is a remarkable finding, since the membrane rearrangement that takes place during capacitation is a key event in the spermatozoa acquisition of their full fertilizing ability. Indeed, male mammalian male gametes gain the ability to interact, recognize and fuse with the homologous oocyte only after they reside in the female genital tract or are exposed to an opportune artificial environment during *in vitro* procedures. In this context, the membrane is either the interface of spermatozoa with the surrounding milieu and the most important signalling system active in messages transduction.

This is the reason why we decided to study the membrane function modifications that ultimately could affect the sperm physiology. Flow cytometry experiments contribute to explain the link between the effects produced by GO on sperm membrane and cytosolic signalling. Overall, it is possible to take some important inferences. The effect on membrane potential and [Ca^2+^]_i_ exerted by BSA and MβCD is very different, either in terms of cellular response and kinetic. In particular, as expected, BSA action is long lasting and favours cell survival, while MβCD has a rapid toxic effect. GO at higher concentrations is detrimental for cell viability and its effect is, in term of length, more similar to MβCD. Nevertheless, at lower concentrations it promotes an increase in [Ca^2+^]_i_ without apparently affecting the membrane potential.

This is a very interesting finding, since the homeostasis of [Ca^2+^]_i_ is one of the key parameters that drives sperm function. It is involved in the control of several function (from motility^50^ to cytoskeleton assembly^51,52^, from lipid remodelling^53^ to apoptosis^54^) and, on the other side, it is controlled by a myriad of factors either at intracellular and extracellular level. Nevertheless, recent reports have described a potential negative effect of GO exposure of sperm cells^55^, somatic stem cells^56^ as well as bacteria^57^. Here, and considering also our functional results obtained in an earlier work, we suggest that the cause could be related to the increase on sperm fertilizing ability, as previously stated by our group^20^. Therefore, since calcium signalling is commonly associated to capacitation, these results seem to reaffirm the role of GO as a facilitator of sperm capacitation.

Finally, as we know, sperm membrane is the interaction site of male gametes with the surrounding environment and, consequently, it is involved in the information exchange that leads the spermatozoa to the acquisition of full fertilizing ability. Several reports agree in describing two different pools of male gametes within the oviduct: one group of spermatozoa present within the lumen, not bound to the epithelial cells and characterized by poor viability and unable to fertilize, and another group, encompassing spermatozoa attached to the OECs with potentially capacitated cells that will be able to fertilize the oocyte^58^. To date, the specific molecular mechanisms involved in such interaction are not completely understood, but it has been supposed that the membrane glycocalyx could be involved^40^. Since the glycocalyx is the first interface with external molecules (such as GO) we carried out a specific set of experiments to exclude any detrimental effect of GO at this level. In particular, we checked if spermatozoa are able to maintain their ability to interact with OECs when they are previously incubated with GO. Our results, as shown in Fig. 7, seem to confirm that the GO treatment does not affect the interaction topology, allowing to hypothesize that this molecule does not compromise the physiological pattern of sperm-epithelial cells recognition and binding, a key step on the road to fertilization.

## Conclusions

We are conscious of the limitations of this study regarding the animal model and the experimental approach adopted. In particular, further experiments are needed to investigate the possible effects of GO on sperm genetic integrity^56^, embryo quality and implantation^18^ and offspring health^59^. Despite this, in our opinion, the present data could have two important applications. On one hand they contribute to shed some light on the interaction between living systems and graphene (and graphene-related materials) at a molecular level. In particular, we found that their possible action in modulating membrane cholesterol content could be useful in engineering cells and/or in designing tools for diagnosis and therapy (the so-called theragnostics). On the other hand, it is possible to hypothesize the potential use of GO and GO-related materials in human and animal assisted reproduction as potential biomaterials, considering that in 2011 more than 600,000 IVF cycles have been performed only in Europe.

## Methods

### Chemicals

Unless otherwise stated, all the chemicals were purchased from Sigma Aldrich (Saint Louis, Missouri). GO aqueous dispersion was purchased from Graphenea (San Sebastian, Spain). The reagents (analytical grade) and HPTLC plates (Kieselgel 60) for lipid analysis were purchased from Merck KGaA (Darmstadt, Germany).

### Preparation of graphene oxide solution

GO aqueous dispersion was obtained by diluting a commercial sample of 4 mg/mL GO, as already described^20^, followed by bath ultrasonication for 10 min (Elmasonic P60H, 37 kHz, 180W) and sterilization for 2 h under UV lamp (Spectronics Spectroline EF 160/C FE, 6W, 50 Hz, 0.17A). GO concentration was checked by UV-vis spectrophotometry (Varian Cary 100 BIO) at λ_max_ 230 nm.

### Preparation and treatment of sperm samples

The preparation of boar semen samples was carried out following an already standardized protocol^29,60,61^. In brief, sperm samples purchased from a specialized company (Suiseme s.r.l., Modena, Italy), after washing, were incubated in a capacitation medium containing TCM199 medium supplemented with 13.9 mM glucose, 1.25 mM sodium pyruvate, 2.25 mM calcium lactate and 1 mM of caffeine (300 mOsm/kg, pH 7.4), at a final concentration of 1 × 10^7^ cells/mL and up to 2 h, at 38.5 °C in 5% CO_2_ humidified atmosphere (Heraeus, Hera Cell). Several samples were subjected to the subsequent analysis: controls (without any treatment), spermatozoa treated with different GO concentrations (5, 2.5, 1.5, 1 and 0.5 µg/mL, added to the capacitating medium), and 2 comparative conditions (0.3% BSA and 1 mM MβCD, added to the capacitating medium). Only samples that showed a motility >90% at the beginning of the incubation were considered for further analysis. All the following experiments were repeated at least three times, on different boars.

### Evaluation of sperm membrane chemical composition after GO exposure

#### Lipid analysis of sperm membrane composition by High-Performance Thin-Layer Chromatography (HPTLC)

Sperm homogenates were sonicated and centrifuged at 800 g for 10 min at 4 °C to eliminate intact cells and nuclear components. Aliquots from the homogenates were used for phospholipids phosphorus determination following the Bartlett procedure. Then, 10 μg of phosphorus from each sample were subjected to lipid extraction according to Tettamanti’s protocol^62^. The lipid extracts in the organic phases were analyzed by HPTLC (High-Performance Thin-Layer Chromatography). In particular, for phospholipid and cholesterol analysis the solvent system was chloroform/methanol/acetic acid/water (60/45/4/2, vol/vol/vol/vol). Phospholipids and cholesterol were visualized on the same HPTLC plate with anisaldehyde reagent (0.5 mL anisaldehyde, 1 mL 97% sulfuric acid in 50 mL glacial acetic acid). After heating at 180 °C for 5 min, the plates were scanned and analyzed with ImageQuant™ TL (GE Healthcare Life Sciences).

#### Gas chromatography analysis of sperm glycerophospholipid fatty acid membrane composition

Sperm samples treated as previously described were washed in DPBS and used for the extraction and transesterification of glycerophospholipids to fatty acid methyl esters (FAME) following a previously described protocol^63^. Briefly, 6 × 10^8^ sperm cells/treatment were resuspended in 200 µL of DPBS, 200 µL of distilled water and 500 µL of methanol and stored at −80 °C until extraction. After thawing, 2100 µL of methanol were added and mixed by vortex, followed by 10 min in a shaker at 250 rpm. After treatment in ultrasonic bath for 5 min, samples were centrifuged for 10 min at 4000 g to separate the cell fragments from the methanolic fraction containing the lipids. Fatty acid esterification reaction was initiated by adding 100 µL of sodium methoxide solution (25wt % in methanol). The reaction was performed at RT and stopped after 4 min by adding 300 uL of methanolic HCl (3 M). FAME were extracted with 1 mL of hexane and recovered in a new vial. The hexane recovered fraction was mixed with 2 mL of 6% potassium carbonate solution followed by 10 min centrifugation at 400 g to separate the phases. The hexane phase was recovered and FAME were dried by a steady nitrogen flow. Extracts were stored at −20 °C until quantification and dissolved in 30 µL of hexane immediately before gas chromatography. FAME were quantified via gas chromatography by a standard procedure using a capillary column ZB-FAME, 30 m × 0.25 mm, film thickness 0.20 µM (Phenomenex, USA) in a 7820 A GC System (Agilent Technologies, Santa Clara, California, EUA). The data was processed using the GC7820 data System (Agilent Technologies, Santa Clara, California, EUA).

### Evaluation of sperm membrane physical composition after GO exposure

#### Evaluation of sperm membrane physical properties by Differential Scanning Calorimetry (DSC) and Thermogravimetrical analysis (TGA)

Once capacitation time was completed, 8 × 10^8^ spermatozoa (8 × 10^8^) were washed with DPBS and membrane enriched fraction (MEF) was isolated according to an adapted protocol^44^. Spermatozoa were resuspended in hypotonic buffer (2 mM Tris, pH 7.2, 12 mM NaCl), then they were sonicated six times for 15 min (with intervals of 1 min between each sonication) and subsequently centrifuged (1600 g for 15 min). The pellet was resuspended in 1 mM potassium phosphate buffer (pH 7.4, 250 mM of sucrose and 0.1 mM of EDTA) and centrifuged (1600 g for 15 min) 3 times in the same buffer. The pooled supernatants were centrifuged at 100.000 g for 1 h and the recovered pellet was lyophilized for 24 h at −20 °C at a pressure of 0.3 hPa. For DSC analysis, MEFs were resuspended in deionized water in a 2:1 ratio. DSC heating thermograms were obtained at a scan rate of 10 °C/min in a range between 15 and 90 °C using a Differential Scanning Calorimeter DSC-7 (Perkin-Elmer Inc., Waltham MA, USA) coupled with a Thermal Analysis Controller TAC 7/DX (Perkin-Elmer Inc., Waltham MA, USA)^64^. The acquired data were analyzed and plotted using the PYRIS Software version 4.02 (Perkin-Elmer Inc., Waltham, MA).

To further characterize the effect of GO on membrane physical properties, we carried out a thermogravimetric analysis. More in detail, MEF from sperm samples, obtained as above described, have been analysed with a Pyris 1 TGA Thermogravimetric Analyzer with the following set up: heat from 30 °C to 800 °C at 20 °C/min; heat from 800 °C to 900 °C at 20 °C/min; nitrogen flow at 40 mL/min.

#### Membrane fluidity assessment by Fluorescence Recovery After Photobleaching (FRAP) analysis

To study the changes in sperm membrane fluidity induced by different treatments, FRAP analysis were carried out, as already described^20^. In brief, washed spermatozoa were stained with the fluorescent probe DilC12(3) perchlorate (ENZ-52206, Enzo Life Sciences, USA), at a concentration of 5 µM in PBS for 20 min (38.5 °C, 5% CO_2_ humidified atmosphere). FRAP experiments were performed at T0 and T2 (after two hours in capacitating medium) using a Nikon A1r laser confocal scanning microscope. Recovery curves were realized and analyzed by using the simFRAP plug-in for Fiji ImageJ^65^, expressing the results as calculated diffusion coefficient (cm^2^/sec).

### Evaluation of sperm membrane biological properties after GO exposure

#### Intracellular calcium concentration and membrane potential assessment by Flow Cytometry

To analyze the effect of GO on calcium intake, spermatozoa were incubated with Fura 2-AM (F1221, Invitrogen) (final concentration of 2 µM) and pluronic acid 0.25%). The effect of GO on plasma membrane potential was monitored by using Bis-oxonol (D8189, Sigma) at the final concentration of 0.02 mM, whereas 7AAD was used as a probe for vitality (50 µg/mL). Co-incubation was carried out for 30 min (38.5 °C, 5% CO_2_, humidified atmosphere) and samples were assessed after 0 h, 30 min, 1 h and 2 h of incubation (T0, T0.5, T1 and T2, respectively).

For each condition and time point, 10.000 events were acquired by flow cytometer (FACSVerse, BD Biosciences - three laser, eight colors configuration, or FACSCanto, BD Biosciences - three laser, eight colors configuration). Each reagent was titrated (8 points titration) under assay conditions and dilutions were established based on achieving the highest signal (Mean Fluorescence Intensity, MFI) for the positive population and the lowest signal for the negative population, and stain indexes were calculated. Instrument performances, data reproducibility and fluorescence calibrations were sustained and checked by the Cytometer Setup & Tracking Beads (BD Biosciences). In order to evaluate non-specific fluorescence, Fluorescence Minus One (FMO) controls were used. Compensation was assessed using CompBeads and FACSuite FC Beads (BD Biosciences) and single stained fluorescent samples. Data were analyzed using FACSuite v 1.0.5 (BD Biosciences) software.

### Evaluation of sperm membrane interaction with swine oviductal epithelial cells (SOEC)

To assess the potential effects of GO on sperm interaction with the oviductal epithelium, swine oviductal epithelial cells (SOEC) were obtained from oviducts collected at a local slaughterhouse and kept to the laboratory in an isothermal contained within 1 h. Then, following a validated protocol and cultured in 3.5 cm Petri dishes with a cover glass at 10^4^ cells/ml for 1 week (38.5 °C, 5% CO_2_ humidified atmosphere). Medium (α-Mem supplemented with 10% FCS, 2% Pen/Strep and 1% ultraglutamine) was refreshed every two days. Once cells reached the confluence, they were coincubated with spermatozoa, previously incubated 30 min under capacitating conditions, with or without GO 1 µg/mL. After 3 washes with DPBS to remove the unbound spermatozoa, the ensemble SOEC-spermatozoa were stained with HOECHST 33342 (9 µM), 5(6)-Carboxyfluorescein diacetate (100 µM) to identify the alive sperm cells and DilC12(3) (5 µM) to delineate the membranes.

## Supplementary information

Results of PCA analysis on lipidomic results

Lipidomic data comparative analysis

FRAP data comparative analysis
