# Supplementary material for: Graphene Oxide increases mammalian spermatozoa fertilizing ability by extracting cholesterol from their membranes and promoting capacitation
Source: Sci Rep. 2019 May 31;9:8155. doi: 10.1038/s41598-019-44702-5 (PMC6544623; doi:10.1038/s41598-019-44702-5)
Supplement: Supplementary file 1 — Results of PCA analysis on lipidomic results [file 41598_2019_44702_MOESM1_ESM.pdf]

Graphene Oxide increases mammalian spermatozoa fertilizing ability by extracting cholesterol from their membranes and promoting capacitation

Nicola Bernabò*[1], Juliana Machado-Simoes[1], Luca Valbonetti[1], Marina Ramal-Sanchez[1], Giulia Capacchietti[1], Antonella Fontana[2], Romina Zappacosta[2], Paola Palestini[3], Laura Botto[3], Marco Marchisio[4,5], Paola Lanuti[4,5], Michele Ciulla[2], Antonio Di Stefano[2], Elena Fioroni[6], Michele Spina[6], Barbara Barboni[1].

PC	% variance
1	99.359
2	0.55079
3	0.072673
4	0.011836
5	0.0037129
6	0.0014187
7	0.00061516
8	0.00019752

Supplementary Information 1.

PCA on lipidomic analysis: % of variance per each Principal Component
