# Supplementary material for: Graphene Oxide increases mammalian spermatozoa fertilizing ability by extracting cholesterol from their membranes and promoting capacitation
Source: Sci Rep. 2019 May 31;9:8155. doi: 10.1038/s41598-019-44702-5 (PMC6544623; doi:10.1038/s41598-019-44702-5)
Supplement: Supplementary file 2 — Lipidomic data comparative analysis [file 41598_2019_44702_MOESM2_ESM.pdf]

Graphene Oxide increases mammalian spermatozoa fertilizing ability by extracting cholesterol from their membranes and promoting capacitation

Nicola Bernabò*[1], Juliana Machado-Simoes[1], Luca Valbonetti[1], Marina Ramal-Sanchez[1], Giulia Capacchietti[1], Antonella Fontana[2], Romina Zappacosta[2], Paola Palestini[3], Laura Botto[3], Marco Marchisio[4,5], Paola Lanuti[4,5], Michele Ciulla[2], Antonio Di Stefano[2], Elena Fioroni[6], Michele Spina[6], Barbara Barboni[1].

Palmitic acid

	T0	T2	BSA	MβCD	GO0,5	GO1	GO1,5	GO2,5	GO5
T0		0,1678	0,8197	0,7809	0,08357	0,2141	0,198	0,0684	0,0225
T2			0,9182	0,9401	1	1	1	0,9999	0,9737
BSA				1	0,7453	0,9563	0,9456	0,6845	0,3605
MβCD					0,7866	0,9703	0,9621	0,7289	0,4002
GO0,5						0,9997	0,9999	1	0,9987
GO1							1	0,999	0,9456
GO1,5								0,9994	0,9563
GO2,5									0,9996
GO5									

Oleic acid

	T0	T2	BSA	MβCD	GO0,5	GO1	GO1,5	GO2,5	GO5
T0		0,02151	1	0,000775	0,004277	0,000359	0,000177	0,000173	0,000173
T2			0,0471	0,7397	0,9957	0,4122	0,01957	0,00025	0,000173
BSA				0,001558	0,00951	0,000596	0,000183	0,000173	0,000173
MβCD					0,9897	0,9996	0,4038	0,002819	0,000174
GO0,5						0,8568	0,09263	0,000559	0,000173
GO1							0,731	0,008713	0,000175
GO1,5								0,2307	0,000266
GO2,5									0,02383
GO5									

Vaccenic

	T0	T2	BSA	MβCD	GO0,5	GO1	GO1,5	GO2,5	GO5
T0		0,08014	0,0865	0,02626	0,01248	0,01056	0,01056	0,01889	0,001902
T2			1	0,9996	0,9873	0,9788	0,9788	0,9975	0,6379
BSA				0,9993	0,9835	0,9733	0,9733	0,9964	0,6134
MβCD					1	0,9999	0,9999	1	0,9177
GO0,5						1	1	1	0,9873
GO1							1	1	0,9929
GO1,5								1	0,9929
GO2,5									0,9593
GO5									

Arachidonic Acid

	T0	T2	BSA	MβCD	GO0,5	GO1	GO1,5	GO2,5	GO5
T0		0,5893	0,995	0,661	0,1453	0,3253	0,6037	0,2259	0,02981
T2			0,9586	1	0,9835	0,9999	1	0,9979	0,6751
BSA				0,978	0,49	0,7824	0,9632	0,6467	0,1393
MβCD					0,9674	0,9994	1	0,994	0,6037
GO0,5						0,9998	0,9809	1	0,994

	T0	T2	BSA	MβCD	GO0,5	GO1	GO1,5	GO2,5	GO5
GO1							0,9998	1	0,9075
GO1,5								0,9973	0,661
GO2,5									0,9674
GO5									

MUFA

	T0	T2	BSA	MβCD	GO0,5	GO1	GO1,5	GO2,5	GO5
T0		0,0324	1	0,001113	0,007428	0,000477	0,000179	0,000173	0,000173
T2			0,0386	0,7428	0,9977	0,4302	0,01887	0,000235	0,000173
BSA				0,001305	0,008896	0,000539	0,00018	0,000173	0,000173
MβCD					0,9843	0,9997	0,3913	0,002178	0,000174
GO0,5						0,8409	0,07879	0,00043	0,000173
GO1							0,7018	0,00631	0,000174
GO1,5								0,1938	0,000249
GO2,5									0,02368
GO5									

PUFA

	T0	T2	BSA	MβCD	GO0,5	GO1	GO1,5	GO2,5	GO5
T0		0,9852	0,9372	1	0,8692	0,9992	0,9929	0,8546	0,347
T2			1	0,9229	0,3418	0,7993	0,673	0,3253	0,06794
BSA				0,8119	0,2246	0,6437	0,5075	0,2124	0,03968
MβCD					0,9651	1	0,9998	0,9585	0,5263
GO0,5						0,9951	0,9995	1	0,9862
GO1							1	0,9935	0,7064
GO1,5								0,9993	0,8274
GO2,5									0,9891
GO5									

SFA/MUFA

	T0	T2	BSA	MβCD	GO0,5	GO1	GO1,5	GO2,5	GO5
T0		0,06482	0,999	0,02612	0,9841	0,02032	0,00746	0,002607	0,001145
T2			0,2053	0,9999	0,3407	0,9995	0,9707	0,7931	0,5427
BSA				0,0912	1	0,07224	0,02757	0,009513	0,003957
MβCD					0,1644	1	0,9993	0,9597	0,8081
GO0,5						0,1324	0,05292	0,01862	0,007711
GO1							0,9999	0,9793	0,8652
GO1,5								0,9998	0,9857
GO2,5									0,9999
GO5									

Peroxidation index

	T0	T2	BSA	MβCD	GO0,5	GO1	GO1,5	GO2,5	GO5
T0		0,9553	0,9165	1	0,9102	0,9998	0,9971	0,8946	0,438
T2			1	0,8781	0,2997	0,7452	0,6165	0,2802	0,06454

BSA	0,8125	0,2391	0,6602	0,5282	0,2226	0,04855
MβCD		0,9713	1	0,9999	0,9638	0,5871
GO0,5			0,9955	0,9995	1	0,9911
GO1				1	0,9936	0,7489
GO1,5					0,9992	0,858
GO2,5						0,9936
GO5						

Supplementary Information 2.

Lipidomic Analysis: Statistical analysis on different classes of lipids: *P* values
