# Supplementary material for: Graphene Oxide increases mammalian spermatozoa fertilizing ability by extracting cholesterol from their membranes and promoting capacitation
Source: Sci Rep. 2019 May 31;9:8155. doi: 10.1038/s41598-019-44702-5 (PMC6544623; doi:10.1038/s41598-019-44702-5)
Supplement: Supplementary file 3 — FRAP data comparative analysis [file 41598_2019_44702_MOESM3_ESM.pdf]

Graphene Oxide increases mammalian spermatozoa fertilizing ability by extracting cholesterol from their membranes and promoting capacitation

Nicola Bernabò*[1], Juliana Machado-Simoes[1], Luca Valbonetti[1], Marina Ramal-Sanchez[1], Giulia Capacchietti[1], Antonella Fontana[2], Romina Zappacosta[2], Paola Palestini[3], Laura Botto[3], Marco Marchisio[4,5], Paola Lanuti[4,5], Michele Ciulla[2], Antonio Di Stefano[2], Elena Fioroni[6], Michele Spina[6], Barbara Barboni[1].

	CTRL	CTRL 2h	GO 0.5 µg/mL	GO 1 µg/mL	GO 1.5 µg/mL	GO 2 µg/mL	GO 5 µg/mL	BMCD	BSA
**CTRL**		0.000944	0.05013	0.000738	0.9694	0.2661	0.1168	0.07763	0.007173
**CTRL 2h**	0.000944		1.03E-05	3.57E-07	0.01343	0.1279	0.06711	0.000284	0.6178
**GO 0.5 µg/mL**	0.05013	1.03E-05		0.03725	0.07524	0.02253	0.004088	0.8344	0.000378
**GO 1 µg/mL**	0.000738	3.57E-07	0.03725		0.0141	0.005184	6.89E-05	0.1572	0.000195
**GO 1.5 µg/mL**	0.9694	0.01343	0.07524	0.0141		0.2733	0.1958	0.03281	0.011
**GO 2 µg/mL**	0.2661	0.1279	0.02253	0.005184	0.2733		0.7999	0.0294	0.182
**GO 5 µg/mL**	0.1168	0.06711	0.004088	6.89E-05	0.1958	0.7999		0.01415	0.1307
**MβCD**	0.07763	0.000284	0.8344	0.1572	0.03281	0.0294	0.01415		0.000448
**BSA**	0.007173	0.6178	0.000378	0.000195	0.011	0.182	0.1307	0.000448	

Supplementary Information 3.

FRAP analysis: Statistical analysis on treatments: *P* values.
